# Cardiac cine with ART for radial parallel imaging reconstruction

**DOI:** 10.1186/1532-429X-15-S1-E34

**Published:** 2013-01-30

**Authors:** Shu Li, Gigi Galiana, Leo Tam, Sebastian Kozerke, Jason P Stockmann, RT Constable, Dana C Peters

**Affiliations:** 1 MRRC, Yale Medical School, New Haven, CT, USA; 2 Institut f. Biomedizinische Technik, ETH Zürich, Zurich, Switzerland

## Background

It was noted early-on that non-Cartesian parallel imaging is achievable by solving the k-space data consistency equation, with inclusion of coil sensitivity profiles [1]. This iterative method which uses gridding is successful [2,3] , as is radial GRAPPA [4]. Here we present the first results of algebraic reconstruction technique (ART) [5,6] which also enforces data consistency. The use of ART with coil-map constraints for radial MRI reconstruction has only recently been described [5] and has not been explored for clinical applications.

## Methods

Short axis cardiac cine data were acquired on a 1.5T Siemens Sonata (Erlangen, Germany), using segmented breath-held 2D radial balanced SSFP with 192 readout points. TR/TE/θ= 2.9/1.5/60°, 36 cm FOV, 930 Hz/pixel, and 4-5 coils. The data was undersampled by factors of R=1 to 8 (192 Np to 24 Np) and reconstructed onto a 192 x 192 matrix. Imaging was performed in five healthy subjects, providing written informed consent. In the iterative ART method [5] each k-space data point is processed, so that the difference between the predicted value, based on the encoding matrix (including coil sensitivities) and the current image estimate, and the actual k-space data point value is inverse encoded and this residual is added to the current image estimate, weighted by λ. Typically, λ=0.08, and number of iterations is 8. No gridding, backprojection or FFTs are used, just the raw encoding matrices based on gradient waveforms, and the coil sensitivities. Coil maps were calculated from the undersampled data sets, by filtered back projection reconstruction of low-passed filtered data. These images were then used for coil sensitivity calculation [7]. Image reconstruction was performed in Matlab R2012a, and was GPU accelerated with a Nvidia GeForce 580, which increased speed by 10, and equivalent image results.

## Results

**Figure 1:**
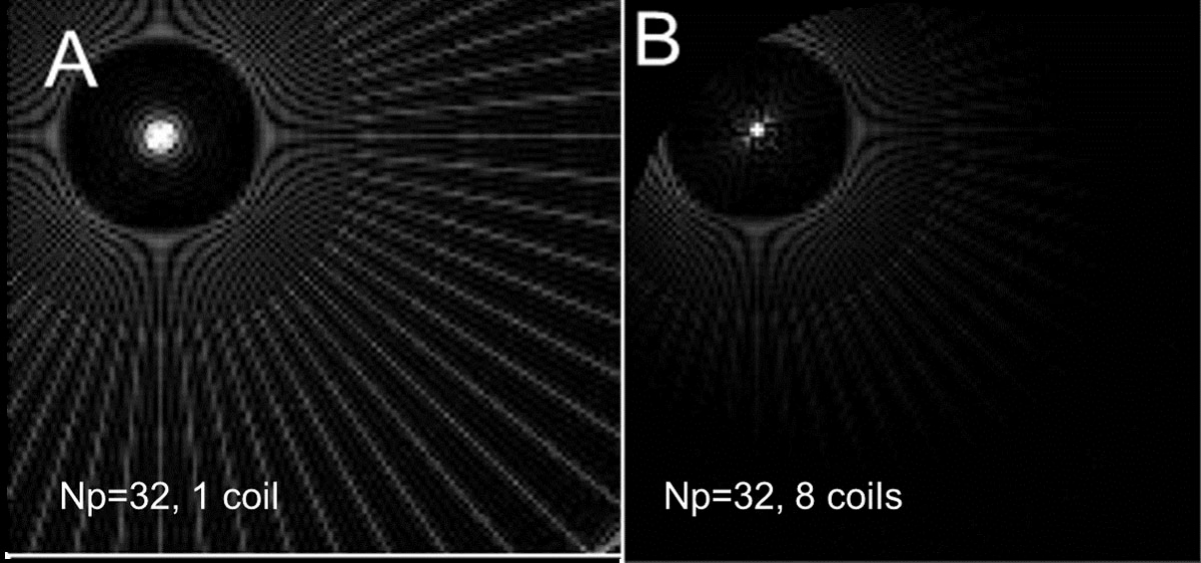
The PSF of a 128 x 32 Np acquisition, reconstructed with ART using A) 1 coil and B) 8 coils circularly arranged. Images are scaled identically (0 to 10%max) to display artifact pattern and central PSF broadening. In B) note the sharper PSF and reduced streaking. In B) the artifact closest to the central point is least suppressed.

**Figure 2:**
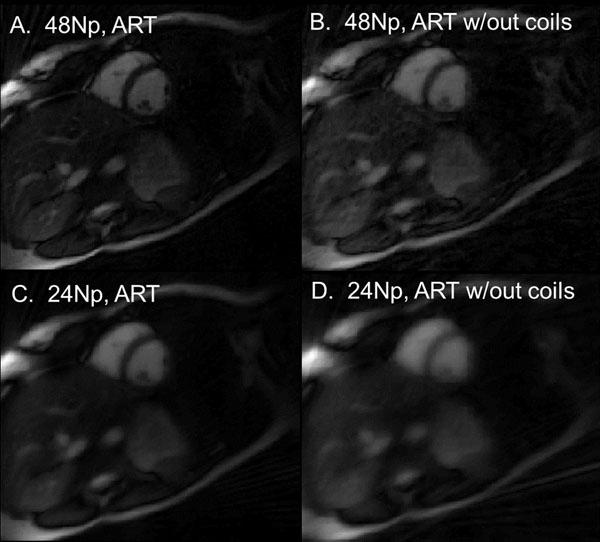
A) 48 Np ART reconstruction exhibits excellent quality, while 48 Np ART without use of coil sensitiviites is blurrier. C) 24 Np ART compared to D) 24 Np ART without coil use.

The point spread functions (PSF) for an off-center point with 1 uniform coil and an circular array of 8 coils (Fig. 1B) are shown for ART technique (32 Np x 128 Nr), demonstrating the reduction in both blurring and artifacts with additional coils. Figure 2 shows the performance of the ART recon for short-axis cine images. Images with 24 and 48 projections are displayed using ART, and ART without coil information (sum of squares). Total reconstruction time (5 coils, 6 iterations, 192 x 48 Np) was 60 seconds.

## Conclusions

ART improves the reconstruction of undersampled radial data, by incorporating coil information, as shown by its first application in cardiac studies.

## Funding

NIH R01 EB 012289.
